# Use of minimally invasive ultrasound transducer during tubular microdiscectomy

**DOI:** 10.3171/2024.1.FOCVID23206

**Published:** 2024-04-01

**Authors:** Michael E. Tawil, Timothy Chryssikos, Abraham Dada, Vardhaan S. Ambati, Mohamed Macki, Samer G. Zammar, Wei Tan, Lee Tan

**Affiliations:** 1 Department of Neurological Surgery, University of California, San Francisco, California;; 2 Department of Neurosurgery, University of Maryland School of Medicine, Baltimore, Maryland; and; 3 Neurological Institute, Cleveland Clinic, Cleveland, Ohio

**Keywords:** intraoperative ultrasound, microdiscectomy, disc herniation

## Abstract

Minimally invasive ultrasound during tubular microdiscectomy is novel. The authors report the technique during surgery for L5–S1 herniated disc. Ultrasound provided real-time visualization of the pathology and neural elements. After discectomy and tactile assessment, ultrasound showed decompression of the thecal sac and traversing nerve root. The patient tolerated the procedure well, with resolution of preoperative pain and strength improvement. Postoperative MRI revealed a residual asymptomatic disc fragment that was retrospectively identified on ultrasonography. Minimally invasive ultrasound could become a useful supplement to direct visual and tactile assessment during tubular microdiscectomy, but further experience with surgical anatomy on ultrasound is required.

The video can be found here: https://stream.cadmore.media/r10.3171/2024.1.FOCVID23206

## Transcript

Here we present the use of a minimally invasive ultrasound transducer during tubular microdiscectomy.

### Disease Background

#### 0:28

Lumbar disc herniation is a common surgical condition that affects up to 5% annually.^1,2^ Indications for urgent surgical treatment include progressive lower-extremity dysfunction and cauda equina syndrome.^1^ Open lumbar discectomy is a well-known and longstanding method for decompression of the neural elements and remains one of the most commonly employed surgical techniques.^3–6^ As an alternative, microdiscectomy has been used for many decades and may result in fewer reoperations with less blood loss, shorter length of stay, and faster return to work.^7,8^ The use of minimally invasive intraoperative ultrasound during tubular microdiscectomy and the description of surgical anatomy using this imaging modality has not been attempted.

### Clinical Presentation

#### 1:17

A 43-year-old female presented with severe lower back and bilateral lower-extremity pain with 4/5 weakness in the right extensor hallucis longus muscle. MRI demonstrated a large disc herniation at the L5–S1 level, resulting in severe compression of the neural elements.

#### 1:38

The rationale and alternatives of the procedure are provided in this slide. The purpose of the surgery was removal of herniated disc material for decompression of the neural elements with minimal disruption of the soft tissues and bony-ligamentous structures. Open lumbar laminectomy was not chosen due to larger size of the incision and more extensive removal of bone and ligament. Irrigating endoscopy was not chosen due to surgeon preference for tubular minimally invasive surgical technique and the large size of the herniated disc on preoperative MRI.

#### 2:14

Risks of the procedure included bleeding, infection, cerebrospinal fluid leak, inadequate decompression, and persistent symptoms. Benefits included improvement or complete resolution of preoperative symptoms with minimal tissue and bony-ligamentous disruption.

### Description of the Setup

#### 2:33

The patient was positioned prone on a Jackson table with Wilson frame, which was cranked to facilitate the decompression. In this slide, we see closer detail of the operative setup after localization of the correct level with C-arm fluoroscopy and placement of the tubular retractor system. Here, an 18-mm × 6-cm tubular retractor has been placed and localized to the L5–S1 level on the right.

#### 3:00

Necessary equipment included C-arm fluoroscopy, minimally invasive tubular retractor system with flex arm for attachment to the bed rail, operative microscope, high-speed burr, microsurgical instruments, and minimally invasive intraoperative ultrasound system. We utilized a linear transducer, 150 mm in length, with a 7 × 6–mm end-fire probe, with a range of 12–15 MHz.

#### 3:28

The key surgical steps are described in this slide. After localization of the L5–S1 level with C-arm fluoroscopy, a small incision is made, and tubular dilators are used with placement of an 18-mm tubular retractor. The position of the tubular retractor is then confirmed with C-arm fluoroscopy. At this point, the operating microscope is brought into the field. After soft-tissue dissection, right L5 laminotomy is carried out with removal of the ligamentum flavum. The minimally invasive intraoperative ultrasound transducer is then placed within the tubular retractor just above the region of interest and images are acquired for review. Following this, herniated disc material is removed. The surgical site is then evaluated for residual disc material using standard tactile assessment with blunt instruments. Following this, the ultrasound system is then utilized once again to evaluate the decompression.

#### 4:29

After soft-tissue dissection using the operative microscope, a high-speed burr is used to create a laminotomy trough to expose the yellow ligament. Soft tissue is then removed with a Kerrison rongeur to reveal the traversing nerve root and lateral thecal sac. At this point, the surgical site is filled with sterile saline, and the minimally invasive ultrasound transducer is situated within the tubular retractor. Short-axis views demonstrate the large disc herniation, which is severely compressing and displacing the thecal sac dorsally. Other anatomical structures visualized include the posterior longitudinal ligament and the anterior spinal column. CSF pulsations from within the compressed thecal sac are seen. Here we see long-axis or sagittal views of the surgical field following the laminotomy and removal of the ligamentum flavum. In these images, we see the traversing nerve root with the offending disc herniation exerting compression on the traversing nerve root. At this point, the thecal sac is mobilized medially, and the disc herniation is brought more clearly into view under the microscope. The site of the annular rupture is seen, and disc material is mobilized with a blunt, right-angled instrument. Herniated disc fragments are then removed with a micropituitary rongeur and other blunt instruments. After removal of several large disc fragments, the plane ventral to the thecal sac is explored with blunt instruments to evaluate for any remaining disc fragments or compressive material. No additional disc material was appreciated using this tactile assessment. The surgical field was again filled with saline, and the minimally invasive intraoperative ultrasound transducer was brought back into the surgical site to evaluate the decompression. Real-time short-axis views demonstrate reestablishment of the patency of the thecal sac with pronounced pulsatility and identification of individual nerve rootlets dispersed freely within the thecal sac. Here we appreciate several other important anatomical landmarks on ultrasound. The remaining lamina is at the uppermost part of the screen. And below this we see the thecal sac and subarachnoid space. Adjacent to the thecal sac we see the traversing nerve root.

### Clinical and Neuroimaging Outcomes

#### 7:02

The patient tolerated the procedure well with no complications. There was near-complete resolution of the patient’s preoperative leg pain, and at discharge, the patient had 5/5 strength in all muscle groups of the lower extremities. Estimated blood loss was 5 ml, and the length of surgery was 45 minutes. A postoperative MRI was acquired to evaluate the decompression. This showed marked improvement in the patency of the central canal. Nevertheless, a small residual disc fragment, ventral to the posterior longitudinal ligament, was appreciated on the postoperative MRI. This residual disc fragment was asymptomatic. This finding on postoperative MRI prompted retrospective assessment of the intraoperative ultrasound images. Looking back, we can appreciate a small focal hyperechoic mass, ventral to a displaced posterior longitudinal ligament. This likely represents the residual herniated disc fragment that was seen on postoperative MRI but was not identified intraoperatively using tactile assessment and was also not appreciated during surgery on the ultrasound images in real time.

#### 8:11

There are several additional points to emphasize for interpretation of this and other intraoperative ultrasound images during tubular microdiscectomy. First, it is important to keep in mind that the ultrasound transducer is oriented to the region of interest in a slightly lateral to medial trajectory in line with the tubular retractor. The second point to emphasize in this still image is the presence of acoustic shadowing on the right side of the screen. This obscures visualization of the most contralateral portions of the thecal sac, posterior, longitudinal ligament, and anterior vertebral column. This acoustic shadowing occurs because the transmitted sound waves of the minimally invasive ultrasound transducer are reflected back by the remaining portion of the bony lamina, which was not removed during laminotomy on the right side. The presence of this acoustic shadowing and the resulting limited visualization of the contralateral aspects of the thecal sac can be considered a limitation of the intraoperative ultrasound technique during minimally invasive laminotomy with microdiscectomy. Nevertheless, despite this acoustic shadowing, we can appreciate that the focal hyperechoic mass situated between the posterior longitudinal ligament and anterior vertebral column is medial to the traversing nerve root and therefore correlates with the central position of the residual disc fragment as seen on postoperative MRI. These teaching points demonstrate that minimally invasive intraoperative ultrasound could have a role to play in the identification of residual disc material during tubular microdiscectomy, and that this modality might serve as a useful supplement to conventional tactile assessment. Additional experience using minimally invasive intraoperative ultrasound during tubular microdiscectomy, awareness of the limitations of the technique, including acoustic shadowing, and correlation with postoperative MRI will help to clarify the interpretation of surgical anatomy as seen with minimally invasive intraoperative ultrasound. This technique could benefit surgeons by providing real-time guidance during minimally invasive tubular decompressive surgery.

## Disclosures

Dr. L. Tan reported being a consultant for Medtronic and Accelus.

## Author Contributions

Primary surgeon: L Tan. Assistant surgeon: Chryssikos. Editing and drafting the video and abstract: all authors. Critically revising the work: Tawil, Chryssikos, Dada, Ambati, Zammar, L Tan. Reviewed submitted version of the work: Tawil, Chryssikos, Dada, Ambati, Zammar, L Tan. Approved the final version of the work on behalf of all authors: Tawil. Supervision: Tawil, Chryssikos, Zammar, L Tan.

## Supplemental Information

### Patient Informed Consent

The necessary patient informed consent was obtained in this study.

## References

[1] Yoon WW Koch J Herniated discs: when is surgery necessary? EFORT Open Rev 2021 6 6 526 530 34267943 10.1302/2058-5241.6.210020 PMC8246101

[2] Frymoyer JW Back pain and sciatica N Engl J Med 1988 318 5 291 300 2961994 10.1056/NEJM198802043180506

[3] Apostolides PJ Jacobowitz R Sonntag VK Lumbar discectomy microdiscectomy: "the gold standard" Clin Neurosurg 1996 43 228 238 9247807

[4] Kim M Lee S Kim HS Park S Shim SY Lim DJ A comparison of percutaneous endoscopic lumbar discectomy and open lumbar microdiscectomy for lumbar disc herniation in the korean: a meta-analysis BioMed Res Int 2018 2018 9073460 30175149 10.1155/2018/9073460 PMC6106715

[5] Chen P Hu Y Li Z Percutaneous endoscopic transforaminal discectomy precedes interlaminar discectomy in the efficacy and safety for lumbar disc herniation Biosci Rep 2019 39 2 BSR20181866 30705086 10.1042/BSR20181866 PMC6379230

[6] Jiang HW Chen CD Zhan BS Wang YL Tang P Jiang XS Unilateral biportal endoscopic discectomy versus percutaneous endoscopic lumbar discectomy in the treatment of lumbar disc herniation: a retrospective study J Orthop Surg Res 2022 17 1 30 35033143 10.1186/s13018-022-02929-5 PMC8760683

[7] Kovačević V Jovanović N Miletić-Kovačević M Standard lumbar discectomy versus microdiscectomy—differences in clinical outcome and reoperation rate Acta Clin Croat 2017 56 3 391 398 29479904 10.20471/acc.2017.56.03.05

[8] Wu X Zhuang S Mao Z Chen H Microendoscopic discectomy for lumbar disc herniation: surgical technique and outcome in 873 consecutive cases Spine (Phila Pa 1976) 2006 31 23 2689 2694 17077737 10.1097/01.brs.0000244615.43199.07

